# Factors affecting stone free rate of primary percutaneous nephrolithotomy on staghorn calculi: a single center experience of 15 years

**DOI:** 10.12688/f1000research.9509.2

**Published:** 2016-09-30

**Authors:** Widi Atmoko, Ponco Birowo, Nur Rasyid

**Affiliations:** 1 Department of Urology, Cipto Mangunkusumo Hospital, Faculty of Medicine, Universitas Indonesia, Jakarta, 10430, Indonesia

**Keywords:** primary percutaneous nephrolithotomy, staghorn calculi, stone-free rate

## Abstract

**Objectives: **Percutaneous nephrolithotomy on staghorn calculi is challenging for urologists because it is difficult to remove all of the stones. The purpose of this study was to evaluate the associated factors of stone-free rate after primary percutaneous nephrolithotomy on staghorn calculi in a large series of patients at a single, tertiary referral, endourologic stone center.

**Methods: **We collected data from medical record between January 2000 and December 2015. A total of 345 primary percutaneous nephrolithotomy procedures were performed for patients with staghorn calculi. This study included both and made no distinction between partial and complete staghorn calculi. Stone-free is defined as the absence of residual stones after undergoing percutaneous nephrolithotomy for the first time. Significant factors from univariate analysis that correlated with stone-free rate after primary percutaneous nephrolithotomy of staghorn stone were further analyzed using multivariate regression analysis.

**Results: **The mean patient age was 52.23±10.38 years. The stone-free rate of percutaneous nephrolithotomy monotherapy was 62.6%. The mean operating time was 79.55±34.46 minutes. The mean length of stay in hospital was 4.29±3.00 days. Using the chi-square test, history of ipsilateral open renal stone surgery ( *p* = 0.01), stone burden ( *p* = < 0.001), and type of anesthesia ( *p* = 0.04) had a significant impact on the stone-free. From multivariate analysis, the history of ipsilateral open renal stone surgery [OR 0.48; 95% CI 0.28-0.81; *p* 0.01] and the stone burden [OR 0.28; 95% CI 0.18-0.45; *p* 0.00] were significant independent risk factors for stone-free.

## Introduction

Staghorn calculus are large and branching kidney stones that occupy a large proportion of the renal pelvis and some or all of the renal calices. Surgical treatment of staghorn calculi involves complete stone removal minimising morbidity. Because untreated staghorn calculus have a tendency to destroy the kidney and cause life-threatening urosepsis, the American Urological Association (AUA) recommends to actively treat all newly diagnosed patients ^1, 2^. In patients with staghorn calculi who are treated conservatively, the mortality rates have been reported to range around 28% to 47.5% ^3– 6^. It is crucial to completely remove all staghorn calculi, because residual stones can form nuclei for stone recurrence (85% recurrence rate) that may lead to infection ^7^.

Percutaneous nephrolithotomy (PCNL) has become the recommended treatment for staghorn calculi as it has stone-free rate three times higher than extracorporeal shock wave lithotripsy (ESWL) and has lower morbidity, shorter length of hospital stay, shorter operating time, and time to return to work faster than open surgery ^1, 8^. Nevertheless, the management of staghorn calculi with PCNL remains challenging. Stone-free rates were lower, complications more frequent, and operative time and hospital stay were longer in patients with staghorn stones compared to nonstaghorn stones ^9^.

However, PCNL is still the mainstay treatment for staghorn calculi, despite the complete removal of staghorn calculi by PCNL being a high skill-demanding surgical procedure and a challenging task for urologists. In this study, we evaluated the stone-free rate and the factors that influence the effectiveness of primary PCNL performed in our national tertiary referral hospital.

## Methods

### Patients

From January 2000 to December 2015, the data from 345 patients with staghorn calculi who had undergone PCNL surgery at the Cipto Mangunkusumo Hospital by one of two surgeons (NR and PB) were reviewed. NR did PCNL surgery since 2000 until now, while PB did PCNL surgery since 2009 until now. This study included both and made no distinction between partial and complete staghorn calculi. Patients who were eligible for the study were adult patients (≥ 18 years old) and those who had PCNL for primary treatment for nephrolithiasis who agreed to enroll by written informed consent. The patients meeting the below criteria were excluded: 1) Patients who had systemic hemorrhagic disease without correction; 2) Patients with severe heart disease and pulmonary incompetence who could not undertake the operation; 3) Uncontrolled diabetes and hypertension patients as well as tuberculosis patients; 4) Patients with renal anatomic malformations, such as horse-shoe and ectopic kidneys, with coexisting staghorn calculi; 5) Lordosis or scoliosis patient who could not tolerate the prone position; 6) Patients who had history of ipsilateral PCNL for secondary or tertiary PCNL. The study protocol was approved by the Ethical Committee, Faculty of Medicine, Universitas Indonesia (No.513/UN2.F1/ETIK/2016).

### Preoperative preparation

Preoperative laboratory examination undertaken included urinalysis, urine culture, serum creatinine, and complete peripheral blood. Plain abdominal radiography of kidneys, ureters, and bladder (KUB) and intravenous urography (IVU) were the primary radiological investigations. Non-contrast computed tomography (NCCT) was performed for patients with high serum creatinine (>1,6 mg/dL) or those allergic to iodinated contrast. Stone burden was assessed pre-operatively by multiplying sum of length and width by means of imaging. Patients with urinary tract infections treated with antibiotics appropriate preoperative urine culture 5 days prior to PCNL. Other patients who had negative urine cultures receiving intravenous antibiotics prior to anesthesia.

### Surgical technique

Following anesthesia, patients were placed in lithotomy position and a 22.5F rigid cystoscope (OLYMPUS) was used to pass a 5F open-end ureteral catheter (Selectip, 62450200; Angiomed, Bard, Murray Hill, NJ) under fluoroscopic guidance, into the renal pelvis, to allow injection of contrast material to delianeate the intrarenal collecting system. A 16F Foley catheter was inserted into the bladder to provide drainage during the procedure and the ureteral catheter was fixed to the Foley catheter. Then the patient was moved to prone position and the side of kidneys to be operated was positioned higher 30°. Percutaneous puncture to gain access to the kidney was done with the help of C-arm control fluoroscopy. Calyx puncture was performed through a superior, media, or inferior, using 18-gauge, diamond-tip needle (Cook Urological, Spencer IN). The needle was positioned so that the target puncture, the needle tip, and the base of the needle was in a position in line. The depth of puncture was controlled using fluoroscopy in the anteroposterior position. After the needle of puncture had been confirmed in the pelvicalyceal system, then a 0.038 guidewire was inserted. After that, the tract was then dilated to 30F using metal dilators (Telescope Bougie Set, 27290A, Karl Storz, Tuttlingen, Germany), fascial dilator and malleable dilators (Amplatz Renal Dilator Set, 075000, Cook Urological, Spencer IN). After inspection by 24-F rigid nephroscope (HOPKINS Wide-Angle Straight Forward Telescope 6°, 27293 AA, Karl Storz, Tuttlingen, Germany), mechanical lithotripsy (Vibrolith, Elmed, Orlando, FL) could be done by breaking the stone. Stone forceps were used to take a hard rock fragments.

### Post operative evaluation

Postoperative imaging were performed 1 or 2 days after PCNL with either Kidney Ureter Bladder (KUB) photos, computed tomography (CT) scan, or antegrade pyelography (APG). Stone-free is defined as the absence of residual stones after undergoing PCNL for the first time. Patients who required additional treatment after their first PCNL, such as secondary PCNL and or ESWL, were automatically excluded from the stone-free group. We also evaluated the transfusion rate and the incidence of postoperative complications, such as infection, urine leakage on operative wounds, intestinal perforation, and bleeding.

### Data analysis

Bivariate analysis was performed by correlating the numerical variables with stone free rates. Those with P value <0.25 were further analyzed with multivariate analysis of logistic regression. Data were analyzed using the Statistical Package for the Social Sciences, version 17 (SPSS Inc., Chicago, IL). The analysis considered significant when P <0.05.

## Results

From January 2000 to December 2015, a total of 345 patients with staghorn calculi had undergone primary PCNL procedures at the Cipto Mangunkusumo Hospital. The mean patient age was 52.23±10.38 years. The stone-free rate of PCNL monotherapy was 62.6%. This value was the result just after the 1st stage of PCNL. The mean operating time was 79.55±34.46 minutes. The mean length of stay in hospital was 4.29±3.00 days. Perioperative transfusions were performed in 11% of patients ( Table 1).

**Table 1.** Patient characteristics.

Variable	Mean ± SD or no. (%) cases
No. patients	345
Age (year)	52.23±10.38
Stone burden (mm ^2^)	51.85±23.54
Body mass index < 25 kg/m ^2^ 25.29.9 kg/m ^2^ ≥ 30 kg/m ^2^	185 (53.6) 98 (28.4) 62 (18.0)
History of ipsilateral renal stone open surgery	85 (24.6)
Calyx target for PCNL access Inferior calyx Other than inferior calyx	312 (90.4) 33 (9.6)
Amount of PCNL access Single Multiple	333 (96.5) 12 (3.5)
Anesthesia Spinal General	281 (81.4) 64 (18.6)
Nephrostomy tube usage Large tube Small tube Tubeless	56 (16.2) 183 (53.0) 106 (30.7)
Stone-free PCNL	216 (62.6)
Operative time (minute)	79.55±34.46
Length of hospital stay (days)	4.29±3.00
Perioperative transfusion	38 (11.0)
Complications Infection Urine leakage at the operative wound Intestinal perforation Bleeding	1 (0.3) 3 (0.9) 1 (0.3) 17 (4.9)

From the univariate analysis, there was significant association between history of ipsilateral renal stone open surgery, stone burden, and type of anesthesia with the stone-free rate (p = 0.01; p < 0.001; p = 0.04, respectively). The univariate analyses are illustrated in Table 2. Stepwise multivariate regression analysis which included variables with p-value < 0.25 showed that the stone burden was the most influential predictor of stone-free (OR 0.28, 95% CI 0.18–0.45, p=0.00) ( Table 3).

**Table 2.** Univariate analysis of factors that associated with the stone-free rate.

Variable	Stone-free rate (%)		*P*
	Stone-free	Residual stone	
Sex Male Female	58.8 41.2	64.3 35.7	0.31*
Age < 65 years ≥ 65 years	87.0 13.0	88.4 11.6	0.72*
Body mass index < 25 kg/m ^2^ 25–29.9 kg/m ^2^ ≥30 kg/m ^2^	50.9 29.6 19.4	58.1 26.4 15.5	0.40*
Stone burden ≤ 52 mm ^2^ > 52 mm ^2^	69.9 30.1	41.1 58.9	0.00*
History of ipsilateral renal stone open surgery Yes No	20.4 79.6	31.8 68.2	0.01*
Calys target for PCNL access Inferior calyx Other calyx	91.2 9.8	89.1 10.9	0.53*
Number of PCNL access Single Multiple	98.1 1.9	93.8 6.2	0.26**
Kidney morphology No hydronephrosis Hydronephrosis	44.4 55.6	41.9 58.1	0.64*
Anestesia General Spinal	16.2 83.8	22.5 77.5	0.15*

*Chi-Square test **Fisher test

**Table 3.** Multivariate analysis (logistic regression model) of factors independently predictive of stone-free rate.

Step	Preoperative factor	Coefficient	p value	OR (CI 95%)
Step 1	History of ipsilateral open renal stone surgery No (reference) Yes	-0.741	0.01	0.48 (0.28–0.80)
	Stone burden ≤ 52 mm (reference) > 52 mm	-1.246	0.00	0.29 (0.18–0.46)
	Anesthesia General (reference) Spinal	-0.263	0.37	1.30 (0.73–2.33)
Step 2	History of ipsilateral open renal stone surgery No (reference) Yes	-0.738	0.01	0.48 (0.28–0.81)
	Stone burden ≤ 52 mm (reference) > 52 mm	-1.267	0.00	0.28 (0.18–0.45)

Raw data for Table 1, Table 2, and Table 3 of 'Factors affecting stone free rate of primary percutaneous nephrolithotomy on staghorn calculi: a single center experience of 15 years’ All the raw data used in univariate and multivariate analises are provided. Click here for additional data file. Copyright: © 2016 Atmoko W et al. 2016 Data associated with the article are available under the terms of the Creative Commons Zero "No rights reserved" data waiver (CC0 1.0 Public domain dedication).

## Discussion

Since the introduction of PCNL to treat kidney stones, there has been a rapid development in techniques and instruments that can be used to treat staghorn calculi and complex stone. In 1983, Clayman *et al.* reported the capability and safety of PCNL in treating staghorn calculi ^10^. Currently, PCNL is the preferred treatment option for patients with staghorn calculi, complex stone, and big stone ^1, 11, 12^. The goal treatment of staghorn calculi is stone-free thoroughly with minimal morbidity ^1, 11^. PCNL in patients with staghorn calculi still represents a procedural challenge, thus requiring the surgeon to perform complete removal of the stone while keeping morbidity to a minimum ^13^.

Stone-free rate after PCNL monotherapy for staghorn calculi is reported to range between 49% to 78% ^13^. In this study, the stone-free rate after PCNL monotherapy was 64.6%. This is higher than the stone-free rate reported by Al - Kohlany *et al.* (49%) ^8^ because they only considered and treated complete staghorn calculi, whereas in this study we included both patients with partial staghorn calculi and complete staghorn calculi and we made no distinction between partial and complete staghorn calculi. Stone-free rate in our study was not very different from the research conducted by El-Nahas *et al.* ^14^ (56.6%) and Desai *et al.* ^9^ (56.9%). They included subject criteria similar to our study, namely the complete and partial staghorn calculi ^14^. However, the stone-free rate of our study was lower than that reported by Soucy *et al.* ^13^ who reported higher stone-free rate (78%). That study incorporated branched stone in just one calyx (borderline staghorn calculi) found in 67% of their patients, so that the majority of patients had a lower burden stone and were easier to treat ^13^.

The duration of the operation is an important factor in determining and comparing various procedural techniques ^15^, as the duration of anesthesia and the risk of pulmonary complications after surgery can indirectly affect the operation outputs (amount of blood loss, decrease of hemoglobin, and blood transfusion requirements) ^16, 17^ and complications ^18, 19^ associated with PCNL. The mean length of surgery in this study was 79.55±34.46 minutes with a median value of 60 (range 20–210) minutes. The mean operating time on research conducted by Huang *et al.* ^20^ was 63.5±11.8 minutes with a range of 29–103 minutes. The duration of operation on that research was shorter because Huang *et al.* did not use a ureteral catheter or balloon catheter before PCNL. According to Huang *et al.*, direct puncture to the stone without previous insertion of ureteral catheter can be done so as to save operating time and reduce complications ^15^.

Potential significant morbidity or even mortality of PCNL have been reported in a large-scale study ^16, 21, 22^. Kidney stone management panel of AUA guidelines mentioned that the staghorn calculi have 7–27% complication and transfusion rate reaching 18% ^1^. Previous studies reported that blood transfusion was needed at 14–24% in PCNL with staghorn calculi, depending on the surgical technique, patient population, indications for transfusion, and the opinion of the surgeon to perform transfusion ^23, 24^. El-Nahas *et al.* reported that the staghorn calculi is a risk factor for the occurrence of severe bleeding in PCNL ^25^. The bleeding complications in our study that required transfusion were lower in numbers than previously reported. As shown in Table 1 and Dataset 1, we observed 4.9% of bleeding cases and 11% cases of perioperative transfusion. Total complications observed in our study amounted to 6.4%.

El-Nahas *et al.* ^26^ found an association between the stone burden (partial and complete staghorn calculi) and secondary calyx stones with a stone-free rate. In our study, no distinction was made between the data entries of complete and partial staghorn calculi but we devided the category of stone burden into two groups, the first group was ≤ 52 mm and the second group was > 52 mm. From our multivariate analysis, we found that the stone burden was associated with the stone-free rate (OR 0.28; 95% CI 0.18-0.45; *p* 0.00). In our study, we didn’t perform S.T.O.N.E nephrolithometry that was found to be the predictor for stone-free rate after PCNL for staghorn stones ^27^. El-Nahas *et al*. ^26^ stated that the stone is branched and secondary stones require multiple access or use flexible nephroscopy to achieve stone-free, but sometimes this technique is not enough. The surgeon must determine whether to increase the number of access PCNL to take the entire residual stone or to treat residual stone with ESWL ^26^. The more the number of PCNL access, the higher the incidence of bleeding complications ^16^.

In this study, we found that history of ipsilateral renal stone open surgery was significantly associated with stone-free rate. This is different from the previous study conducted by Kurtulus *et al.* ^28^ that compared patients who undergone PCNL for the first time with patients who had previous history of open renal stone surgery. In patients who have a history of open renal stone surgery, infundibulum stenosis, perinephric fibrosis, bowel displacement, and incisional hernia are the major factors that should be taken into account by the surgeon ^29, 30^. As long as the safety rules are strictly followed, PCNL can still be performed with minimal complication and high success rates despite the technical and access difficulties encountered in secondary or tertiary cases due to anatomic positional differences of the kidney and fibrosis as mentioned by Kurtulus *et al*. ^28^. In their study, the residual stone rate wasn’t significantly different between patients who had previous history of ipsilateral open renal stone surgery and patients who undergone PCNL for the first time (5% vs 3%, p>0.05). Kurtulus *et al.* had difficulty in dilating percutaneous tract in patients with history of ipsilateral open renal stone surgery. With the help of newly developed high-pressure balloons, assistance of fascial dilators, or by mechanical dilators, difficulty in establishing access may be overcomed ^28^. In some other studies, it had been reported that open stone surgery can increase PCNL failure rate ^31^, while others showed that previous open stone surgery does not affect PCNL outcome ^32– 34^.

The type of anesthesia was not significantly associated with stone-free rate in our multivariate analysis. This finding was in accordance with other studies. Astram *et al*. compared 220 PCNL procedures using general anesthesia and 540 PCNL using spinal anesthesia. They found the stone-free rate in the general anesthesia group was 71.37%, similar to the spinal anesthesia group 72.97% (p > 0.05) ^35^. Kuzgunbay *et al.* ^36^ and Tangpaitoon *et al.* ^37^ also found that combined spinal-regional anesthesia is a feasible technique in PCNL operations because the efficacy and safety were not affected compared to PCNL with general anesthesia. Selection of anesthesia is important because it can affect the patient's postoperative recovery and a consideration for the urologist to discharge a patient from the hospital in a safe condition as soon as possible ^38^. In our study, the majority of PCNL was performed under spinal anesthesia (81.4%) and no conversion from spinal to general anesthesia was recorded. It was found that the use of spinal anesthesia can reduce the need for PCNL postoperative analgetic, decrease nausea ^39^, and the patient can cooperate when operation being held ^36^. General anesthesia on the other hand, may increase complications in PCNL when the patient changes position ^40^. Additionaly, performing PCNL on staghorn calculi under general anesthesia can induce diluted anemia, hypothermia, higher blood loss, as well as the possibility of fluid absorption and electrolyte imbalance ^38^. In short, lower dose of analgesia demand, duration of surgery, well-maintained hemodynamic stability during and after operation with faster patient recovery shows the promising aspect of spinal anesthesia to be virtually used in most PCNL procedures ^41^.

This study bears the common problems of retrospective studies, including selection bias and missing of important clinical data, like partial or complete staghorn stone. The results reported here are different from those published in the study conducted by El-Nahas *et al* ^26^. They found that independent risk factors for residual stones were complete staghorn calculi and presence of secondary calyceal stones (relative risks were 2.2 and 3.1, respectively). In our study, we didn’t distinct between partial and complete staghorn calculi and this type of analysis could not be done. Besides that, stone free status was a primary endpoint. However, it was evaluated by either plain KUB radiograph, CT scan, or antegrade pyelography. There would be bias on these images since it could probably missed 3–4 mm residual fragment on a plain KUB film. It could be difficult to evaluate stone free status accurately with plain KUB film in an early postoperative period since fluid leakage around the kidney may obscure residual fragments. In addition, the low metabolic evaluation in patients is a weakness of this study because the stone analysis and the metabolic tests are not used routinely on all patients. No follow-up data collection on secondary treatment (such as ESWL, ureterorenoscopy (URS), and secondary PCNL) is also a shortcoming of this study because from those data we ccould analyse the effectiveness of combination therapy with ESWL, secondary PCNL effectiveness rate, and other therapies.

## Conclusions

Percutaneous nephrolithotomy is the mainstay for treating staghorn calculi. History of ipsilateral renal stone surgery and stone burden are prognostic factors determining stone clearance after PCNL on staghorn stones.

## Data availability

The data referenced by this article are under copyright with the following copyright statement: Copyright: © 2016 Atmoko W et al.

Data associated with the article are available under the terms of the Creative Commons Zero "No rights reserved" data waiver (CC0 1.0 Public domain dedication). 

F1000Research: Dataset 1. Raw data for Table 1, Table 2, and Table 3 of 'Factors affecting stone free rate of primary percutaneous nephrolithotomy on staghorn calculi: a single center experience of 15 years’, 10.5256/f1000research.9509.d134117 ^42^

## Consent

Written informed consent to participate in the study and publish clinical data was obtained by the patients.

## Abbreviations & acronyms

APG: Antegrade Pyelography

AUA: American Urological Association

ESWL: Extracorporeal Shock Wave Lithotripsy

IVU: Intravenous Urography

KUB: Kidneys, Ureters, and Bladder

NCCT: Non Contrast Computed Tomography

PCNL: Percutaneous Nephrolithotomy

URS: Ureterorenoscopy
