# Supplementary material for: Factors affecting stone free rate of primary percutaneous nephrolithotomy on staghorn calculi: a single center experience of 15 years
Source: F1000Res. 2016 Sep 30;5:2106. Originally published 2016 Aug 30. [Version 2] doi: 10.12688/f1000research.9509.2 (PMC5031123; doi:10.12688/f1000research.9509.2)
Supplement: Peer review history

# Peer review: Referee response for version 2

**DOI:** 10.5256/f1000research.10400.r16734

**Published:** 2016-10-07

**Authors:** Manint Usawachintachit

**Affiliations:** 1 Department of Urology, University of California San Francisco, San Francisco, CA, USA

I have read this submission. I believe that I have an appropriate level of expertise to confirm that it is of an acceptable scientific standard.

# Peer review: Referee response for version 1

**DOI:** 10.5256/f1000research.10243.r15959

**Published:** 2016-09-16

**Authors:** Manint Usawachintachit

**Affiliations:** 1 Department of Urology, University of California San Francisco, San Francisco, CA, USA

The authors present a retrospective study looking for associated factors of stone free rate following PCNL on staghorn stones. The primary endpoint was stone free status evaluated at an early postoperative period. It’s interesting data in that the number of procedures is high (345).

 Overall, the paper was written concisely with a good methodology in research design and statistical analysis. I would recommend this article is indexed after addressing some issues enumerated below: 

**Introduction**

- No specific comments.

 

**Material and Methods**

- This is a retrospective study over a time period of 15 years. The authors didn’t mention about the surgeon’s level of experience so I wonder if there will be any effect on stone free rate by the surgeons’ learning curve.
- Stone free status was a primary endpoint and a main focus in this study. However, it was evaluated by either plain KUB radiograph, CT scan, or antegrade pyelography. Could there be any bias based on these images, since we probably missed 3-4 mm residual fragment on a plain KUB film?  
- Additionally, it could be difficult to evaluate stone free status accurately with plain KUB film in an early postoperative period. Fluid leakage around the kidney may obscure residual fragments.   

**Results**

- In Table 2, data regarding Number of PCNL access was duplicated with the above row.
- How would nephrostomy tube size correlate to stone free status? I think the tube size most likely depends on surgeon’s preference and other factors such as bleeding or requirement for postoperative drainage.    

 

**Discussion**

- No specific comments.

I have read this submission. I believe that I have an appropriate level of expertise to confirm that it is of an acceptable scientific standard.

# Peer review: Referee response for version 1

**DOI:** 10.5256/f1000research.10243.r15960

**Published:** 2016-09-09

**Authors:** Frederick Singer

**Affiliations:** 1 John Wayne Cancer Institute, Providence Saint Johns Health Center, Santa Monica, CA, USA

This is a large retrospective analysis of the recurrence rate over 15 years of staghorn calculi after removal of staghorn calculi by percutaneous nephrolithotomy which examines the factors which appear to influence the recurrence rate. Univariate analysis indicated that a history of ipsilateral open renal stone surgery, stone burden and the type of anesthesia were significant factors in determining a stone-free outcome whereas multivariate analysis eliminated the type of anesthesia as a risk factor. The authors pointed out that they did not have adequate data concerning the metabolic factors that could have made a significant contribution to the recurrence rate. Nevertheless this is an otherwise worthwhile contribution to understanding the appropriate treatment of staghorn calculi.

I have read this submission. I believe that I have an appropriate level of expertise to confirm that it is of an acceptable scientific standard.
